# Clinical analysis of women with ovarian pregnancy: a retrospective case–control study

**DOI:** 10.1186/s12884-022-05099-8

**Published:** 2022-10-13

**Authors:** Hongyi Li, Yu Liu, Yang Yang, Xia Zhao, Xiaorong Qi

**Affiliations:** Department of Gynecology and Obstetrics, Development and Related Disease of Women and Children Key Laboratory of Sichuan Province, Key Laboratory of Birth Defects and Related Diseases of Women and Children, Ministry of Education, West China Second University Hospital, Sichuan University, 20 South Renmin Road, Block 3, Chengdu City, 610041 Sichuan Province People’s Republic of China

**Keywords:** Ovarian pregnancy, Tubal pregnancy, Clinical complications, Risk factors, Laparoscopy

## Abstract

**Background:**

To address the clinical features and potential risk factors of ovarian pregnancy (OP).

**Methods:**

In this retrospective case–control study performed in West China Second University Hospital from March 17, 2005 to December 8, 2018, 146 OP patients were selected as a case group, 292 patients with tubal pregnancy (TP) and 292 women with intrauterine pregnancy (IUP) were matched as controls at a ratio of 1:2:2.

**Results:**

When compared with TP patients, OP patients tend to have worse clinical complications (hemorrhagic shock (7.41% vs 2.89%), rupture of pregnancy sac (54.07% vs 37.78%), hemoperitoneum (363.1 ± 35.46 ml vs 239.3 ± 27.61 ml) and increased need for emergency laparotomy (9.60% vs 3.97%) at an early gestational age. Assisted reproductive technology (ART) (adjusted OR1 2.08, 95%CI 1.04 to 4.18; adjusted OR2 2.59, 95%CI 1.25 to 5.37) and intrauterine contraceptive device (IUD) use (adjusted OR1 2.19, 95%CI 1.10 to 4.36; adjusted OR2 2.77, 95%CI 1.74 to 5.71) may be risk factors for ovarian ectopic pregnancy as compared to the control groups of TP and IUP patients.

**Conclusions:**

OP patients tend to have more severe clinical complications and this study has identified ART and IUD use as potential risk factors for OP. Results of this study may contribute to improve the understanding of OP and promote early surgical intervention.

**Supplementary Information:**

The online version contains supplementary material available at 10.1186/s12884-022-05099-8.

## Background

Primary ovarian pregnancy (OP), which refers to the implantation of the fertilized ovum in the ovary, is one of the rarest forms of non-tubal ectopic pregnancy [1]. Its incidence following natural conception ranges from 1/2,000 to 1/60,000 pregnancies and it accounts for about 0.5 ~ 3% of all extra-uterine pregnancies [2–4]. Since the first case of OP was reported by Saint Maurice in 1682, its incidence has been on the rise due to increased awareness of this disease and availability of sensitive assays for serum beta-human chorionic gonadotropin (β-hCG) detection as well as the development of transvaginal ultrasound [5, 6].

Approximately 91% of OP cases terminate in the first trimester and are often misdiagnosed as tubal pregnancy (TP), hemorrhagic ovarian cysts or ruptured corpus luteum prior to surgery because of their similar signs and symptoms (e.g. abdominal pain, vaginal bleeding, circulatory collapse and ruptured focus) [7]. More seriously, OP usually results in rupture of gestational sac and hemoperitoneum because of increased vascularity of ovarian tissue, making it a life-threatening gynecological emergency. Preoperative diagnosis of OP remains challenging and exact diagnosis depends largely on histologic findings. Therefore, counselling for high-risk patients before conception and better understanding on its risk factors can aid early diagnosis of OP and reduce the associated morbidities.

However, there is limited data available for systematic analysis of OP’s clinical manifestations and possible risk factors, due to the low incidence of the disease. Traditional etiological factors of tubal pregnancies such as previous pelvic infection or endometriosis have not been found to associate with risk factors for ovarian pregnancies [8], and the exact risk factors for OP remain to be ascertained. In this study, we retrospectively reviewed medical records of 146 OP cases that were diagnosed and treated in West China Second University Hospital during the last 13 years. The aim of this study was to compare clinical characteristics between OP and TP patients and to examine possible risk factors associated with OP with control groups comprising TP and IUP.

## Methods

This study conducted a retrospective review of patients that are roughly distributed with a ratio of 1:2:2 in the OP (*n* = 135), TP (*n* = 277) and IUP (*n* = 285) groups, from March 17, 2005 to December 8, 2018 in West China Second University Hospital in Chengdu, Sichuan, China (Patients who did not undergo surgery or had incomplete information were excluded. Data collection profile of this study can be found in the Supplementary material). All methods were carried out in accordance with relevant guidelines and regulations. Data were extracted from patients' files and it was approved by Ethics Committee of the hospital. Women who were intraoperatively diagnosed with OP on pathological examination [9] were classified within the case group. TP patients were randomly selected from the in-patient department, who had a pathological diagnosis of ectopic pregnancy in the fallopian tube. Patients with IUP were from maternity ward and IUP were confirmed with ultrasonography and serum β-hCG levels.

We reviewed the electronic medical records to analyze the sociodemographic characteristics (including age, body mass index (BMI), marital status, occupation, and smoking); clinical features (clinical manifestations at the time of presentation in hospital, eg, abdominal pain, vaginal bleeding and hemorrhagic shock; auxiliary examination results before surgery, eg, gestational age (wk) at operation date, initial β-hCG level (IU/mL), preoperative hemoglobin (Hb) concentration (g) and sonographic findings of the pelvic); and risk factors of ectopic pregnancy (including number of previous induced abortions and parity; previous ectopic pregnancy, infertility and mode of pregnancy; surgical history of caesarean section, adnexal surgery and previous appendectomy; and contraceptive use). Operation methods, intraoperative findings (including site of ectopic pregnancy, pelvic adhesion, the amount of hemoperitoneum detected intraoperatively and rupture of the ectopic gestational sac), operating time, total intraoperative blood loss, blood transfusion and hospital stays were also recorded.

All patients had received an ultrasound examination before surgery and the serial serum concentrations of β-hCG were recorded. The amount of hemoperitoneum was preliminarily estimated by the depth of pelvic effusion in preoperative ultrasound scan. During the operation, hemoperitoneum was measured by subtracting the total volume of saline used for the irrigation from the total fluid volume aspirated.

### Statistical analysis

Student t test was used for continuous variables, while Chi-square test was used for categorical variables. A multivariate logistic regression model was applied to identify independent factors associated with OP. Statistical analysis of data was performed using SPSS ver. 13.0 for Windows (SPSS Inc., Chicago, IL, USA) with *p*-values < 0.05 considered statistically significant.

## Results

During the study period (2005 to 2018), 158,762 pregnancies and 4674 ectopic pregnancies (EP) occurred at our hospital and ovarian pregnancy comprised 3.12% (146/4674) of all EPs, which was equivalent to 1 case per 1087 pregnancies. The sociodemographic characteristics of the three groups were summarized in Table 1. All three groups were matched in terms of age and BMI. There were no significant difference among the three groups in terms of occupation and smoking. However, significant differences were found in marital status (*p* = 0.01).

**Table 1:** Sociodemographic characteristics of enrolled participants

	**OP group (*n* = 135)**	**TP group (*n* = 277)**	**IUP group (*n* = 285)**	***p* value**
	**n (%)**	**n (%)**	**n (%)**	
Age(yrs)	28.3 ± 5.2	29.4 ± 5.8	29.1 ± 6.3	0.49
≤ 24	36 (26.67)	69 (24.91)	71 (24.91)	
25–29	42 (31.11)	86 (31.05)	87 (30.53)	
30–34	43 (31.85)	72 (25.99)	87 (30.53)	
≥ 35	14 (10.37)	50 (18.05)	40 (14.04)	
BMI (kg/m2)				0.21
Mean ± SD	21.33 ± 2.50	21.90 ± 2.75	22.41 ± 2.49	
Marital status				**0.01**
Married	111 (82.22)	243 (87.73)	263 (92.28)	
Unmarried	24 (17.78)	34 (12.27)	22 (7.72)	
Occupation				0.42
Employed	43 (31.85)	106 (38.27)	99 (34.73)	
Unemployed	92 (68.15)	171 (61.73)	186 (65.26)	
Smoking				0.18
Non-smoking	134 (99.26)	270 (97.78)	274 (96.14)	
Smokers	1 (0.74)	7 (2.22)	11 (3.86)	

*OP* Ovarian pregnancy, *TP* Tubal pregnancy, *IUP* Intrauterine pregnancy

Boldface indicates *p* < 0.05

Tables 2 and 3 show the association between OP risk and relevant patient clinical history when compared with control groups, including reproductive history, gynecological history, previous abdominal surgical history, and contraceptive use. There were no significant differences among 3 groups in terms of parity, previous cesarean section and appendectomy history. Then we used multivariate logistic regression analysis to compare the potential risk factors of OP, the final model of which included the following variables: induced abortion, history of previous ectopic pregnancy, infertility, adnexal surgery, mode of pregnancy and contraceptive use. When TP women were used as controls, the OR of OP among women who had abortion once was lower than in those who had no previous abortion (adjusted OR1 0.44, 95% CI 0.22 to 0.89). In addition, the ORs of OP were significantly lower in women who had a history of ectopic pregnancy (adjusted OR1 0.32, 95% CI 0.17 to 0.61), infertility (adjusted OR1 0.33, 95% CI 0.15 to 0.71) and a history of previous adnexal surgery (adjusted OR1 0.42, 95% CI 0.23 to 0.77). In contrast, women who underwent assisted reproductive technology (ART) and used intrauterine device (IUD) were at a higher risk of OP (adjusted OR1 2.08, 95% CI 1.04 to 4.18; adjusted OR1 2.19, 95% CI 1.10 to 4.36) than those who did not. Further, the incidence of OP was significantly higher than that of IUP when ART was used instead of conceiving naturally and IUD was implemented instead of applying no contraceptive measures at all (adjusted OR2 2.59, 95% CI 1.25 to 5.37; adjusted OR2 2.77, 95% CI 1.74 to 5.71). Table 4 outlines the clinical features of patients in the OP and TP groups. Complaints of abdominal pain at presentation (*p* = 0.11) and initial serum β-hCG level (*p* = 0.89) were similar between the two groups. However, women with OP were less likely to initially present with vaginal bleeding than those with TP (*p* < 0.01). In addition, hemorrhagic shock (*p* = 0.04), rupture of pregnancy sac (*p* = 0.02), and emergency laparotomy (*p* = 0.04) were more frequent in the OP group than in the TP group. Moreover, earlier gestational age at operation date (*p* < 0.01) and lower Hb level prior to surgery (*p* = 0.01) were observed in the OP group. In terms of sonographic findings, there was no significant difference in the appearance of ectopic gestational sac between the two groups. Specifically, the volume of pelvic effusion in the OP group, determined by a typical sonographic parameter termed as extensive hemoperitoneum, was larger than the TP group (*p* < 0.01).

**Table 2:** Reproductive, gynecological and previous surgical history of all enrolled participants

	**OP group (*n* = 135)**	**TP group (*n* = 277)**	**IUP group (*n* = 285)**	***P* value**
	**n (%)**	**n (%)**	**n (%)**	
Reproductive history				
Induced abortion				**0.04**
0	65 (48.15)	108 (38.99)	146 (51.23)	
1	40 (29.63)	76 (27.44)	67 (23.51)	
2	18 (13.33)	47 (16.97)	34 (11.93)	
≥ 3	12 (8.89)	46 (16.61)	38 (13.33)	
Parity				0.51
0	73 (54.07)	144 (51.99)	139 (48.77)	
1	50 (37.04)	117 (42.24)	128 (44.91)	
≥ 2	12 (8.89)	16 (5.78)	18 (6.32)	
Previous ectopic regnancy				** < 0.01**
No	122 (90.37)	208 (75.09)	257 (90.18)	
Yes	13 (9.63)	69 (24.91)	28 (9.82)	
Previous infertility				** < 0.01**
No	127 (94.07)	232 (83.75)	261 (91.58)	
Yes	8 (5.93)	45 (16.25)	24 (8.42)	
Previous abdominal surgery				
Cesarean section				0.71
No	100 (74.07)	214 (77.26)	213 (74.74)	
Yes	35 (25.93)	63 (22.74)	72 (25.26)	
Adnexal surgery				** < 0.01**
No	120 (88.89)	213 (76.90)	264 (92.63)	
Yes	15 (11.11)	64 (23.10)	21 (7.37)	
Appendectomy				0.70
No	130 (96.30)	263 (94.95)	269 (94.39)	
Yes	5 (3.70)	14 (5.05)	16 (5.61)	
Mode of pregnancy				**0.02**
Natural pregnancy	118 (87.41)	259 (93.50)	270 (94.74)	
ART	17 (12.59)	18 (6.50)	15 (5.26)	
Contraceptive experience				** < 0.01**
None users	94 (69.63)	241 (87.00)	245 (85.97)	
Intrauterine device	19 (14.07)	10 (3.61)	14 (4.91)	
Oral contraceptive pills	5 (3.70)	6 (2.17)	10 (3.51)	
Condoms	17 (12.59)	20 (7.22)	16 (5.61)	

*OP* Ovarian pregnancy, *TP* Tubal pregnancy, *IUP* Intrauterine pregnancy, *ART* Assisted reproductive technology

Boldface indicates *p* < 0.05

**Table 3:** Multivariate logistic regression analysis of potential risk factors for OP

	**Adjusted OR1** **(95% CI)**	***P*1 value**	**Adjusted OR2** **(95% CI)**	***P*2 value**
	**OP vs TP**		**OP vs IUP**	
Induced abortion				
0	Ref		Ref	
1	0.44 (0.22 to 0.89)	**0.02**	0.71 (0.35 to 1.45)	0.34
2	0.50 (0.24 to 1.04)	0.06	0.53 (0.25 to 1.13)	0.10
≥ 3	0.68 (0.30 to 1.57)	0.37	0.24 (0.25 to 1.42)	0.60
Previous ectopic pregnancy				
No	Ref	** < 0.01**	Ref	0.95
Yes	0.32 (0.17 to 0.61)		0.98 (0.49 to 1.95)	
Previous infertility				
No	Ref		Ref	
Yes	0.33 (0.15 to 0.71)	** < 0.01**	0.69 (0.30 to 1.57)	0.37
Adnexal surgery				
No	Ref		Ref	
Yes	0.42 (0.23 to 0.77)	** < 0.01**	1.57 (0.78 to 3.15)	0.20
Mode of pregnancy				
Natural pregnancy	Ref		Ref	
ART	2.08 (1.04 to 4.18)	**0.04**	2.59 (1.25 to 5.37)	**0.01**
Contraceptive experience				
None users	Ref		Ref	
Intrauterine device	2.19 (1.10 to 4.36)	**0.03**	2.77 (1.74 to 5.71)	** < 0.01**
Oral contraceptive pills	0.45 (0.16 to 1.22)	0.12	0.78 (0.30 to 2.07)	0.62
Condoms	1.02 (0.26 to 3.94)	0.98	2.13 (0.60 to 7.58)	0.25

*OP* Ovarian pregnancy, *TP* Tubal pregnancy, *IUP* Intrauterine pregnancy, *ART* Assisted reproductive technology

Boldface indicates *p* < 0.05

**Table 4:** Comparison of clinical features between the OP and TP groups

	**OP group (*n* = 135)**	**TP group (*n* = 277)**	***P* value**
	**n (%)**	**n (%)**	
Abdominal pain			0.11
Yes	116 (85.93)	219 (79.06)	
No	19 (14.07)	58 (20.94)	
Vaginal bleeding			** < 0.01**
Yes	46 (34.07)	225 (81.23)	
No	89 (65.93)	52 (18.77)	
Hemorrhagic shock			**0.04**
Yes	10 (7.41)	8 (2.89)	
No	125 (92.59)	269 (97.11)	
Rupture of pregnancy sac			**0.02**
Yes	73 (54.07)	114 (37.78)	
No	62 (45.93)	163 (62.22)	
Emergency laparotomy			**0.04**
Yes	12 (9.60)	11 (3.97)	
No	113 (90.40)	266 (96.03)	
Gestational age at operation date (wk)			** < 0.01**
Mean ± SD	5.84 ± 1.89	7.02 ± 1.93	
Initial β-hCG level (IU/mL)			0.89
Mean ± SD	3.41 ± 0.58	3.39 ± 0.64	
Hb prior to surgery (g)			**0.01**
Mean ± SD	107.8 ± 18.96	113.2 ± 15.23	
Sonographic findings			
Depth of pelvic effusion (Mean ± SD mL)	2.581 ± 1.87	1.905 ± 1.76	** < 0.01**
Showing the ectopic gestational sac			
Yes	109 (80.74)	238 (85.93)	0.20
No	26 (19.26)	39 (14.08)	

*OP* Ovarian pregnancy, *TP* Tubal pregnancy

Boldface indicates *p* < 0.05

Four hundred five patients (132 from the OP group and 273 from the TP group) underwent laparoscopic surgery and only 7 patients (3 from the OP group and 4 from the TP group) underwent laparotomy because of circulatory collapse or severe abdominal adhesion. Table 5 shows that there were no significant differences in the site of ectopic pregnancy sac (left or right) (*p* = 0.29), type of surgery (*p* = 0.69), pelvic adhesion (p = 0.10), blood transfusion (*p* = 0.24), and days of hospital stay (*p* = 0.76). In contrast, the operating time (*p* = 0.04) was longer and volume of total blood loss during operation (*p* = 0.04) was larger in the OP group. There was a significant difference in the amount of hemoperitoneum between the two groups (*p* < 0.01), which was consistent with the preoperative ultrasound findings.

**Table 5:** Surgical outcomes

	**OP group (*n* = 135)**	**TP group (*n* = 277)**	***P* value**
	**n (%)**	**n (%)**	
Site of ectopic pregnancy			0.29
Left	66 (48.89)	151 (54.51)	
Right	69 (51.11)	126 (45.49)	
Type of surgery			0.69
Laparoscopy	132 (97.78)	273 (98.56)	
Laparotomy	3 (0.22)	4 (1.44)	
Method of lesion resection			
Resection of OP	135 (100.00)	0 (0.00)	
Salpingectomy	0 (0.00)	160 (57.76)	
Salpingostomy	0 (0.00)	117 (42.24)	
Amount of hemoperitoneum (mL)			** < 0.01**
Mean ± SD	363.1 ± 35.46	239.3 ± 27.61	
Pelvic adhesion			0.10
No adhesion	29 (21.48)	41 (14.80)	
Tubo-ovarian adhesion	13 (9.63)	48 (17.33)	
Cul-de-sac adhesion	93 (68.89)	188 (67.78)	
Blood transfusion			0.24
Yes	7 (5.19)	7 (2.53)	
No	128 (94.81)	270 (97.47)	
Total blood loss (ml)			**0.04**
Mean ± SD	67.05 ± 17.64	31.42 ± 3.99	
Operating time (min)			**0.04**
Mean ± SD	58.25 ± 2.05	52.50 ± 1.82	
Hospital stay (days)			0.76
Mean ± SD	3.76 ± 1.76	3.82 ± 1.45	

*OP* Ovarian pregnancy, TP Tubal pregnancy

Boldface indicates *p* < 0.05

## Discussion

OP is a relatively uncommon variant of ectopic pregnancy and few studies with a decent number of OP cases have been reported. During the study period (2005 to 2018), 4674 EPs occurred at our hospital and ovarian pregnancy comprised 3.12% (146/4674) of all ectopic pregnancies. To the best of our knowledge, the present study included the largest number of OP cases compared to previous investigations. It is likely that the frequency of OP is underestimated since some early ovarian pregnancies has been reported to be suspected tubal pregnancies that are treated medically, without laparoscopic validation [10]. This underestimation is balanced by a more awareness to the possibility of an OP and more careful histologic examination of the ovarian tissues.

An untreated ovarian pregnancy causes potentially fatal intra-abdominal bleeding and thus may become a medical emergency. The unusual site and rarity of OP lead to a more complex clinical course, beginning with the difficulty in making an early and accurate diagnosis, resulting in a possible unpredictable outcome and a life-threatening situation if the ovary ruptures [11, 12]. Patients with OP usually have similar symptoms to those encountered in tubal ectopic pregnancy. As in our study, the typical symptoms are abdominal pain and vaginal bleeding. Circulatory collapse was present in 10 (7.41%) of 135 OP patients in our study. However, in a case–control study conducted from 2005 to 2014, the incidence of circulatory collapse was reported to be 15.49% (11 of 71 OP patients) [13]. Although the reported incidence of circulatory collapse varies among different studies, the hemorrhagic shock rates are generally higher in the OP group than in the TP group. The natural history of OP indicates that the gestational sac usually ruptures within a certain period of time after development. The rupture of an ectopic gestational sac was significantly more common in the OP group (54.07% VS 37.78%) and the gestational age at operation date was earlier in the OP group (5.84 ± 1.89 VS 7.02 ± 1.93 wk). Interestingly, our study didn’t find significant difference in serum β-hCG levels in the two groups. Oliver R et al. [14] suggested ovarian ectopic were associated with low (< 1000 IU/L) serum β-hCG levels and a case report of a ruptured primary OP has also been published [15]; Other studies indicates that OP patients tend to have higher β-hCG levels than women with tubal pregnancy [13, 16]. Our study also found a higher incidence of emergency laparotomy and hemorrhagic shock in OP patients than in TP patients. Further, the amount of hemoperitoneum observed during the operation was significantly higher in the OP group than in the TP group. These findings collectively indicate that OP patients tend to have a poorer prognosis than TP patients.

Seinera et al. [8] speculated that traditional risk factors for tubal ectopic pregnancy were not relevant risk factors for ovarian pregnancies. In contrast, some researchers believe that increased OP risk may be associated with factors such as endometriosis [17], previous adnexal surgeries, previous infectious diseases, history of infertility [18], in vitro fertilization and embryo transfer (IVF-ET) [19–21], polycystic ovarian syndrome and intrauterine device (IUD) use [22]. In the present study, we found that ART treatment was significantly more common in OP patients than in TP and IUP patients, suggesting ART as an OP risk factor. Several theories have been proposed to explain why ART is a risk factor for OP: (1) large volume and high pressure of culture medium injected during embryo transfer, difficult ET and manipulation with tissue forceps [23]; (2) reverse migration of the transferred embryos toward the fallopian tube and implantation in the ovary after deep deposition in the uterine cavity [24, 25]; (3) high estrogen stimulates uterine contraction and gonadotropin stimulates ovarian enlargement, thus contributing to the development of OP [26, 27]. Although all these mechanisms explain how OP occurs after IVF-ET, the mechanism underlying the higher OR of OP than TP in women who underwent ART remains elusive and requires further study.

Besides, as an effective method of contraception, IUD is frequently mentioned as the etiologic factor of OP [28, 29]. In accordance with previous reports, our study also found the use of IUD is related to the occurrence of OP. In the present study, 14.07% (19/135) patients in the OP group, 3.61% patients (10/277) in the TP group and 4.91% (14/285) patients in the IUP group were current users of IUD, indicating that women using IUD are more likely to have OP. The main mechanism of action of the IUD is the production of continuous sterile inflammatory reaction in the uterine cavity due to the foreign body. Some researchers presumed that the presence of an IUD in situ may increase host susceptibility to infection, thus increasing the risk of pelvic inflammatory disease (PID). One study suggested that IUD reduced uterine implantation by 99.5%, tubal implantation about 95%, but has little protective effect against OP [22]. An explanation for this discrepancy might be the fact that IUD reduces intrauterine implantation but do not have the same protective effect against OP. We have to understand that current assumptions on the risks of ovarian pregnancy are mainly based on individual case series and retrospective case control studies with limited OP subjects. Whether these factors play aetiological roles in the increase of OP occurrence remains debated, and the exact risk factors for OP remain to be ascertained.

Preoperative diagnosis of ovarian pregnancy remains challenging. The four criteria described by Otto Spiegelberg for diagnosing ovarian pregnancy are generally established by careful histologic examination from all surgical materials of ectopic pregnancies and cannot be established by ultrasonography. With the availability of more sensitive methods for hCG detection and improvements in ultrasonography, early diagnosis of OP cases has become possible. Ultrasound scans can detect gestational sacs at 5.5 to 6 weeks of gestation and beyond [30]. Ultrasound imaging features of OP include: (1) there is no intrauterine gestational sac and the endometrium is thickened; (2) unruptured OPs have characteristic solid hyperechoic rings or masses, within which distinct blood flow signals and sometimes even embryonic and fetal heartbeats can be observed; (3) no characteristic ultrasonogram was detected in ruptured OPs which were all diagnosed as ruptured ectopic pregnancy or corpus luteum and is difficult to clearly distinguish by ultrasound [31]. New criteria combining biochemical and ultrasound findings have been proposed: (1) serum β-hCG level ≥ 1000 IU/L and no gestational sac in the uterus at vaginal ultrasonography; (2) ovarian implication should be confirmed by surgical exploration, with bleeding, visualisation of chorionic villi or presence of an atypical cyst on the ovary; (3) normal tubes; and (4) absence of serum β-hCG after treatment of the ovary [32]. These new diagnostic criteria should lead to more accurate diagnosis of OP as well as reveal the true prevalence when highly suspected cases do not meet the four criteria of Spiegelberg.

Laparoscopy has emerged as a simple method for confirming the location of the pregnancy directly and has been accepted as the preferred exact diagnostic and therapeutic method for ectopic pregnancy [33]. In view of concerns about future fertility, the most common surgical treatment of OP is laparoscopic wedge-shaped resection of pregnancy lesions and preserving healthy ovarian tissue [21]. In our study, this conservative surgical management allowing preservation of the ovary and reproductive capability was performed in most OP patients. The follow-up β-hCG level decreased to normal range in about two weeks, within one month, when tracked postoperatively. It is also noteworthy that the outcome of subsequent pregnancy is successful, with a low rate of subsequent ectopic pregnancy. Successful treatment of an ovarian pregnancy with methotrexate (MTX) has also been reported and such medical treatment has the advantage of less invasiveness than surgery [34, 35]. However, in most cases its use is limited since the potential risk of massive bleeding, in which case a subsequent diagnostic laparoscopy is required.

### Strengths and limitations

The strength of our study is that we compared TP and IUP with OP to analyze the clinical characteristics and potential risk factors for ovarian pregnancy. Secondly, to the best of our knowledge, the present study included the largest number of OP cases compared to those have been previously reported. Our study is also subject to several limitations. First, we only collected data from our hospital. It may hide information because of the limited sample size and lead to false negative results. Large sample study is required to further investigation. Next, due to the lack of follow-up data, our findings do not allow for an analysis of the effect of ovarian pregnancy history on the outcome of repeat pregnancies in patients.

## Conclusion

Early diagnosis of ovarian pregnancy is necessary in order to avoid serious complications. This study compared clinical manifestations of OP and TP and showed that OP patients were more likely to have worse clinical complications (hemorrhagic shock, rupture of gestational sac, hemoperitoneum and need for emergency laparotomy) at an early gestational age. Our findings also indicated that ART and current IUD use were risk factors of OP. For patients whose gestational sac is not detected in the uterus or the fallopian tubes, these risk factors and clinical features seem to have a high predictive value and may contribute to early suspicion of OP, thereby optimizing its clinical management.

## Supplementary Information

**Additional file 1.** Schematic diagram of data collection.

## Data Availability

The datasets used and analyzed during the current study available from the corresponding author on reasonable request.

## Abbreviations

OP: Ovarian pregnancy
β-hCG: β-Human chorionic gonadotropin
TP: Tubal pregnancy
IUP: Intrauterine pregnancy
ART: Assisted reproductive technology
IUD: Intrauterine contraceptive device
EP: Ectopic pregnancy
BMI: Body mass index
IVF-ET: In vitro fertilization and embryo transfer
PID: Pelvic inflammatory disease
MTX: Methotrexate
