# Supplementary material for: Clinical analysis of women with ovarian pregnancy: a retrospective case–control study
Source: BMC Pregnancy Childbirth. 2022 Oct 13;22:768. doi: 10.1186/s12884-022-05099-8 (PMC9560037; doi:10.1186/s12884-022-05099-8)
Supplement: Supplementary file 1 — Additional file 1. Schematic diagram of data collection. [file 12884_2022_5099_MOESM1_ESM.docx]

**Table1: Schematic diagram of data collection**

Women with OP (n=146)

3 with no surgery

8 with incomplete information

case group (n=135)

Women with TP (n=292)

Women with IUP (n=292)

11 with no surgery

4 with incomplete information

TP control group (n=277)

7 with incomplete information

IUP control group (n=285)

A matched case-control study

OP: ovarian pregnancy TP: tubal pregnancy IUP: intrauterine pregnancy
